# Challenges in Pediatric Endocrinology

**DOI:** 10.3390/children10111757

**Published:** 2023-10-30

**Authors:** Zvi Laron

**Affiliations:** Endocrine and Diabetes Research Unit, Schneider Children’s Medical Center, Tel Aviv University, Petah Tikva 49200, Israel; laronz@clalit.org.il

As the Section Editor-in-Chief, it is my pleasure to introduce the new section of *Children* devoted to pediatric endocrinology. Its scope is to publish original academic articles, reviews, and special editions on achievements in ongoing basic and clinical research in endocrinology, diabetes, metabolism, genetics and to review the effects of new drugs designed to prevent or treat diseases in those fields.

Pediatric endocrinology became a separate discipline in the 1940s due to the work of Lawson Wilkins in Baltimore and Nathan Talbot in Boston [1], who also published the first textbooks on this topic in 1950 [2] and 1952, respectively [3].

Since then, technological advancements, using biochemical and genetic methods, have enabled the disclosure of the molecular mechanisms of many clinically recognized diseases and the discovery of new ones [4,5,6]. During this period, the norms for growth and the basis of pediatric endocrinology were designed [7].

However, despite the substantial knowledge accumulated, there remain many challenging questions to be answered. Below are a few examples.

Many mechanisms causing abnormal growth, such as the etiology of idiopathic short stature [8,9,10], overgrowth [11], or growth without growth hormone [12], are still uncertain. Of great concern are the continuous increases in the worldwide incidences of Type I diabetes [13], obesity, and Type II diabetes [14].

Preventive interventions in Type I childhood diabetes have failed so far, and there is no cure [15]. The increasing numbers of youth in many countries with gender dysphoria or sex development present many diagnostic and therapeutic problems [16,17,18].

Newly designed growth-stimulating drugs such as long acting growth hormone preparations or novel approaches for the treatment of achondroplasia [19,20] will need close follow-up for their efficacy and early or late adverse effects.

Our new section will strive to follow new research in all aspects of pediatric endocrinology and disseminate the results to pediatric endocrinologists, neonatologists, geneticists, and pediatricians in practice.
